# Athletics for Girls

**Published:** 1921-05-21

**Authors:** 


### THE PUBLIC WELL-BEING.

### Athletics for Girls.

The usual kind of newspaper controversy has been started by the violent and sweeping condemnation of girls' games made at a conference of head-mistresses and women "who have made a study of physical culture." A resolution was passed protesting against the present system of physical education for girls as being injurious to future generations. But the mistake made by these educationists and their friends was so to overstate their case as to play directly into the hands of opponents of their theories. To assert, for instance, that the modern physically trained girl is a degenerate, that feet are the only part of a woman's body that need development, that Swedish gymnastics are a subtle form of pro-German propaganda introduced into this country by way of Sweden in order to diminish the British population, is to reduce the discussion to the level of the ridiculous. Of course, the reply of the physical culturists has been prompt and emphatic. According to the secretary of one of the clubs at the London School of Medicine for Women, the students at that institution indulge in strenuous sports with the sanction and encouragement of authority, especially rowing. All the girls who take up rowing or sculling are medically examined, and cases of ill-health or strain are unknown among the students."

Among medical women, those whose opinions were invited were generally in favour of athletics for girls. Dr. Alice Benham expressed a typical view when she declared that girls are much healthier and fitter for playing games and taking vigorous exercise, as they are encouraged to do at the big schools. It would be difficult, as she rightly points out, to refute the sweeping statements made as to the ill-effects of exercise on the future generation without comparing statistics over a long period. As far as her own experience in maternity work goes, her worst cases have been among young women of the very feminine type, or "blue stockings" who have spent their lives in study, without playing any strenuous games. It would be interesting to know what are the views on this subject of medical practitioners generally, and a good deal of valuable information might be obtained from other points of view from the thousands of school-mistresses in all parts of the country in whose schools physical training has become a regular part of the curriculum. Sir George Newman, as Chief Medical Officer under the Board of Education, has made almost a speciality of his insistence on physical training; and his theory of the importance of outdoor games and physical exercises in the child-life of both sexes appears to be strongly supported by the great body of school medical officers. This theory is now being gradually put into practice everywhere, as the means offer; and, though it is too early to be at all dogmatic as to the results so far as girls are concerned, or to differentiate very accurately between games and exercises that are more or less suitable for girls, it is probably true to say that the value of sport for girls depends very largely on the discretion and knowledge of those who are responsible for whatever system is employed.

It is quite obvious that excessive indulgence in violent exercise over a prolonged period might have serious results in the upbringing of a young girl. It is also obvious that a form of athleticism suitable for one type of girl is unsuitable for another. But precisely the same thing might be said of boys. The people who are creating a wholly false atmosphere with regard to this question are the sex extremists on one side who argue as if there were no fundamental differences to be considered in the physique of the two sexes, and the extremists on the other side who imagine that there is no game which a boy plays well that a girl can play without harm to herself, or ought to be allowed to try to play.

